# Successful toric intraocular lens implantation in a patient with induced cataract and astigmatism after posterior chamber toric phakic intraocular lens implantation: a case report

**DOI:** 10.1186/1752-1947-6-109

**Published:** 2012-04-16

**Authors:** Kazutaka Kamiya, Akio Nakamura, Hideyuki Miyake, Hiroyuki Nishimoto, Kimiya Shimizu

**Affiliations:** 1 Department of Ophthalmology, University of Kitasato School of Medicine, 1-15-1 Kitasato, Minami, Sagamihara, Kanagawa, 252-0374, Japan; 2 Nakamura Eye Clinic, 7-9, Katsuta, Chuo, Hitachinaka, Ibaraki, 312-0045, Japan

## Abstract

**Introduction:**

We report the case of a patient in whom simultaneous toric phakic intraocular lens removal and phacoemulsification with toric intraocular lens implantation were beneficial for reducing pre-existing astigmatism and acquiring good visual outcomes in eyes with implantable collamer lens-induced cataract and astigmatism.

**Case presentation:**

A 53-year-old woman had undergone toric implantable collamer lens implantation three years earlier. After informed consent was obtained, we performed simultaneous toric implantable collamer lens removal and phacoemulsification with toric intraocular lens implantation. Preoperatively, the manifest refraction was 0, -0.5 × 15, with an uncorrected visual acuity of 0.7 and a best spectacle-corrected visual acuity of 0.8. Postoperatively, the manifest refraction was improved to 0, -0.5 × 180, with an uncorrected visual acuity of 1.2 and a best spectacle-corrected visual acuity of 1.5. No vision-threatening complications were observed.

**Conclusion:**

Toric intraocular lens implantation may be a good surgical option for the correction of spherical and cylindrical errors in eyes with implantable collamer lens-induced cataract and astigmatism.

## Introduction

The Visian Implantable Collamer Lens (Visian ICL, STAAR Surgical, Nidau, Switzerland), a posterior chamber phakic intraocular lens (IOL), has been reported for superiority not only in postoperative visual performance compared to laser *in situ *keratomileusis but also for safety and effectiveness in the correction of moderate to high ametropia [1-3]. In addition, this surgical procedure is largely reversible and the lens exchangeable with another lens, unlike laser *in situ *keratomileusis, even when unexpected refractive changes occur after surgery.

Recently, toric ICL implantation has also been demonstrated to be an effective means for the correction of high myopic astigmatism [3-6]. However, there are ongoing concerns about the development of lens opacity after both non-toric and toric ICL implantation, because of the close proximity of the ICL to the crystalline lens, and about endothelial cell loss after surgery. In light of the prevalence of this surgical procedure, it is clinically important to assess the prognosis of patients with a developing cataract after ICL implantation.

Simultaneous ICL removal and phacoemulsification with conventional IOL implantation has been reported to be safe and effective with predictable refractive results, and thus yield a high level of satisfaction for these patients [7-9]. However, toric ICL removal and non-toric IOL implantation may lead to an increase in astigmatism in toric ICL-implanted eyes, because these eyes have some corneal astigmatism, and this astigmatism remains after these combined procedures.

Reducing this pre-existing astigmatism and acquiring good visual outcomes are essential to minimize spectacle dependence and maximize subsequent patient satisfaction, even for patients with ICL-induced cataracts. Nevertheless, to the best of our knowledge, there have been no previous case reports on the management of ICL-induced cataracts and astigmatism by phacoemulsification with toric IOL implantation. We present here a case report indicating that simultaneous toric ICL removal and phacoemulsification with toric IOL implantation was beneficial for acquiring a good visual outcome in an eye with an ICL-induced cataract and astigmatism.

## Case presentation

A 53-year-old woman, who had undergone bilateral toric ICL implantation for treatment of high myopic astigmatism in both eyes three years earlier, visited our hospital complaining of blurred vision in her left eye. In that eye, the manifest refraction was 0, -0.5 × 15, with an uncorrected visual acuity (UCVA) of 0.7 and a best spectacle-corrected visual acuity (BSCVA) of 0.8. The ICL vault (the distance between the posterior surface of the ICL and the anterior surface of the crystalline lens) was 0.16 mm; the keratometry, 42.8/45.2D × 95; the endothelial cell density, 2924 cells/mm^2^; and the central corneal thickness, 529 μm (Table 1).

**Table 1:** Preoperative and postoperative data

	Preoperative	Postoperative (one month)
Mean refraction	0, -0.5 × 15	0, -0.5 × 180
Uncorrected visual acuity	0.7	1.2
Best spectacle corrected visual acuity	0.8	1.5
Keratometry (D)	42.8/45.2 × 95	42.8/45.5 × 100
Corneal astigmatism (D)	2.4	2.7
Endothelial cell density (cells/mm^2^)	2,924	2,801
Central corneal thickness (μm)	529	530
Intraocular pressure (mmHg)	12	10

**Figure 1:**
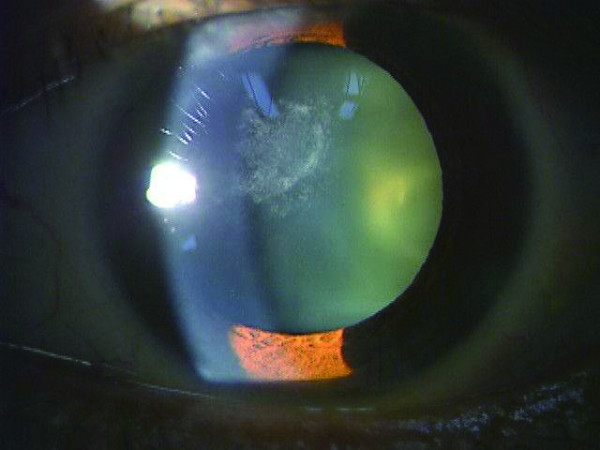
**Preoperative slit-lamp examination disclosed a marked anterior subcapsular cataract reaching as far as the axial area**.

Slit-lamp examination showed a marked anterior subcapsular cataract reaching into the visual axis (Figure 1). The remainder of the examination was unremarkable.

After informed consent was obtained, we performed simultaneous toric ICL removal and phacoemulsification with toric IOL implantation. IOL spherical power calculations were performed by the SRK-T formula using the axial length measured by A-scan ultrasonography (US-800, Nidek, Aichi, Japan), and keratometric readings measured with an autorefractometer (RK-5, Canon, Tokyo, Japan). IOL cylinder power and the alignment axis were calculated using a Web-based toric IOL calculator program [10], along with the default surgically induced astigmatism, which the software considered as 0.5D. To control for potential cyclotorsion, the horizontal axis was marked preoperatively using a slit-lamp in a supine position. For ICL removal and phacoemulsification with toric IOL implantation, a 3.0 mm temporal clear corneal incision at a site identical to the original incision was created after topical anesthesia. After the introduction of viscoelastic material (Opegan; Santen, Osaka, Japan) into the anterior chamber, the proximal haptics of the ICL were dislocated, grasped with forceps, and extracted from the anterior chamber through the incision. Then, standard phacoemulsification was performed by capsulorrhexis, nuclear and cortex extraction, and toric IOL (SA60AT5, power +9.5D; Alcon Laboratories Inc., Fort Worth, Texas, USA) implantation using the same 3.0 mm incision. After surgery, our patient received steroidal (0.1% betamethasone; Rinderon, Shionogi, Tokyo, Japan), antibiotic (0.5% levofloxacin; Cravit, Santen) and non-steroidal diclofenac sodium 0.1% (Diclod, Wakamoto, Tokyo, Japan) medications, which were topically administered four times daily for one month and then the dose steadily reduced.

On day 1, our patient's UCVA was 1.2. One month after surgery, the mean refraction was 0, -0.5 × 180, and she had a UCVA of 1.2 and a BSCVA of 1.5, and the keratometry was 42.8/45.5D × 100. The surgically induced corneal astigmatism was 0.54D × 126. Her endothelial cell density was 2801 cells/mm^2 ^(Table 1), and our patient was very satisfied with the postoperative visual outcomes. No vision-threatening complications occurred, including rotation of the IOL, and the manifest refraction and her visual acuity remained stable throughout the six-month follow-up period.

## Discussion

As far as we can ascertain, this is the first case report on the management of ICL-induced cataract and astigmatism by phacoemulsification with toric IOL implantation. Recently, toric ICL implantation has also been demonstrated to be effective for the correction of high myopic astigmatism [3-6]. However, the possible risk of cataract formation was not negligible, especially in older patients or those with high myopia [11,12] who underwent not only non-toric but also toric ICL implantation. It is of clinical importance to reduce pre-existing astigmatism and to acquire good visual outcomes, particularly in toric ICL-implanted eyes, because toric ICL-implanted eyes have a certain amount of astigmatic error and deteriorated quality of vision, and because patients who have undergone refractive surgery typically desire the best uncorrected visual outcomes.

In recent years, toric IOL implantation has been reported to be effective for the correction of spherical and cylindrical errors in cataractous eyes having some corneal astigmatism. The Acrysof toric IOL has provided excellent rotational stability in comparison with other toric IOLs, and so we selected this IOL for our patient to acquire good UCVA. Although toric multifocal IOL implantation is not available in Japan, it may also be a viable surgical option for patients with cataracts and astigmatism who demand good visual acuity without glasses for both far and near.

With regard to the predictability of these surgical procedures, Bleckmann and Keuch stated that refractive error in the eyes has never exceeded the value of 1D, independent of the initial refraction or degree of hyperopia or myopia [7]. Morales *et al. *reported that the percentage of eyes within ± 1.0D of the targeted correction was 71.4% [8]. We have also previously demonstrated that, three months after surgery, the percentage of eyes within ± 0.5D of the targeted correction was 80%, and within ± 1.0D of the targeted correction was 90% [9]. In this study, we measured the axial length by A-scan ultrasonography without any correction, and used the SRK-T formula to calculate spherical IOL power for this patient after toric ICL implantation. Hoffer *et al. *reported that the greatest difference in A-scan axial length measurements occurs with a silicone IOL in an eye with very high hyperopia, and that the least difference will occur with a collamer lens (ICL) in an eye with very high myopia [13]. Morales *et al. *also stated that the difference between the axial length measurements before and after ICL implantation was small [8]. These findings may partly account for the higher predictability of these combined procedures.

## Conclusion

The findings of our case report suggest that the use of a toric IOL may be an alternative for the treatment of spherical and cylindrical errors in eyes with an ICL-induced cataract and astigmatism. We also suggest that standard biometric measurements can be obtained and used in effective IOL power calculations in the event of a cataract after ICL surgery, regardless of whether or not the ICL was spherical or toric. Considering that toric ICL-implanted eyes have a certain amount of corneal astigmatism, which can decrease uncorrected acuity and deteriorate quality of vision, the preliminarily results are encouraging. A further study in a large series of patients should be conducted to confirm these preliminary findings.

## Abbreviations

BSCVA: best spectacle-corrected visual acuity; ICL: implantable collamer lens; IOL: intraocular lens; UCVA: uncorrected visual acuity.

## Consent

Written informed consent was obtained from the patient for publication of this case report and any accompanying images. A copy of the written consent is available for review by the Editor-in-Chief of this journal.

## Competing interests

KS is a consultant to STAAR Surgical. The remaining authors declare that have no competing interests.

## Authors' contributions

KK, AN, HM, HN and KS treated the patient and in doing so acquired the case data; they were also involved with drafting of the manuscript. All authors read and approved the final manuscript.
